# Graphic Warning Labels and the Cost Savings from Reduced Smoking among Pregnant Women

**DOI:** 10.3390/ijerph14020164

**Published:** 2017-02-08

**Authors:** John A. Tauras, Richard M. Peck, Kai-Wen Cheng, Frank J. Chaloupka

**Affiliations:** 1 Department of Economics, University of Illinois at Chicago, Chicago, IL 60637, USA; rmpeck@uic.edu (R.M.P.); kwcheng@uic.edu (K.-W.C.); fjc@uic.edu (F.J.C.); 2 National Bureau of Economic Research (NBER), Cambridge, MN 02138, USA; 3 Tobacco Center of Regulatory Science, Georgia State University, Atlanta, GA 30303, USA; 4 Institute of Health Research and Policy, University of Illinois at Chicago, Chicago, IL 60637, USA

**Keywords:** prenatal smoking, low birth weight, cost estimations, graphic warning labels

## Abstract

*Introduction:* The U.S. Food and Drug Administration (FDA) has estimated the economic impact of Graphic Warning Labels (GWLs). By omitting the impact on tobacco consumption by pregnant women, the FDA analysis underestimates the economic benefits that would occur from the proposed regulations. There is a strong link between the occurrence of low birth weight babies and smoking while pregnant. Low birth weight babies in turn generate much higher hospital costs than normal birth weight babies. This study aims to fill the gap by quantifying the national hospital cost savings from the reductions in prenatal smoking that will arise if GWLs are implemented in the U.S. *Data and Methods:* This study uses several data sources. It uses Natality Data from the National Vital Statistics System of the National Center for Health Statistics (NCHS) in 2013 to estimate the impact of prenatal smoking on the likelihood of having a low-birth-weight baby, controlling for socio-economic and demographic characteristics as well as medical and non-medical risk factors. Using these estimates, along with the estimates of Huang et al. (2014) regarding the effect of GWLs on smoking, we calculate the change in the number of LBW (low birth weight) babies resulting from decreased prenatal smoking due to GWLs. Using this estimated change and the estimates from Russell et al. (2007) and AHRQ (2013) on the excess hospital costs of LBW babies, we calculate cost saving that arises from reduced prenatal smoking in response of GWLs. *Results and Conclusions:* Our results indicated that GWLs for this population could lead to hospital cost savings of 1.2 billion to 2.0 billion dollars over a 30 year horizon.

## 1. Introduction

Maternal smoking during pregnancy is strongly linked to infant low birth weight (LBW; 2500 grams or less). Because maternal smoking during pregnancy is a significant factor for LBW babies and treating LBW babies is expensive, maternal smoking imposes high medical costs. Russell et al. (2007) [1] estimate hospital costs for the first year of life for uncomplicated births as well as preterm and low birth weight babies. These costs include delivery, transfers, and readmissions for the first year of life. Restating Russell et al. (2007) estimates to 2015 dollars using the medical care component of the consumer price index, the excess hospitalization cost for a low birth weight baby (1500–2500 g) relative to an uncomplicated birth is $23,746 and for an extremely low birth weight baby (<1000 g) relative to an uncomplicated birth is $106,448. Unfortunately, Russell et al. (2007) did not calculate the hospital costs separately for very low birth weight babies (1000–1499 g). However, we can compute this figure utilizing Healthcare Cost and Utilization Project data provided by the Agency for Healthcare Research and Quality (AHRQ, 2013) [2]. Again re-expressing this estimate to 2015 dollars using the medical care component of the consumer price index, the excess hospitalization cost for a very low birth weight baby relative to an uncomplicated birth is $84,627.

The U.S. Food and Drug Administration (FDA), which was charged with regulating tobacco products by the 2009 Family Smoking Prevention and Tobacco Control Act (FSPTCA), proposed and finalized a regulation requiring large graphic warning labels (GWLs) to be added to cigarette packs sold in the U.S. Although court challenges by tobacco companies prevented FDA from implementing the rule, FDA has said it remains committed to implementing its responsibilities under the FSPTCA, which include revising cigarette warning labels. Thus, estimating the various public health benefits that would result from GWLs remains important from a policy perspective [3].

Cigarette package warnings that are graphic, larger, and more comprehensive in content are effective in communicating the health risks of smoking [4]. These graphic images are more likely than text-only warning labels to promote smoking-related knowledge [5,6]. The adoption of GWLs in Canada has reduced overall smoking prevalence [7,8]. These results have also been supported by other studies [8]. 

In this paper, we examine potential hospital cost savings generated by the reduction of low birth weight babies that arise from graphic warning labels. First, we provide new estimates of the link between cigarette consumption by pregnant women and their baby’s birth weight. Our estimates use the U.S. 2013 Natality File, a comprehensive, large data set of nearly 4 million observations. Controlling for a myriad of confounding factors including mother and baby characteristics, characteristics of the delivery, socio-economic factors, and sources of payment, we estimate the impact maternal smoking has on the probabilities of giving birth to a low birth weight baby, a very low birth weight baby, and an extremely low birth weight baby.

Using these new estimates, along with the Huang et al.’s (2014) [7] estimates of the effect of GWLs on smoking in Canada, we calculate the decline in the number of LBW babies in the U.S. that would result from adding GWLs to cigarette packs in the U.S. To our knowledge, Huang et al. (2014) is the only study published to date that quantifies the effect of GWLs on smoking prevalence. Using this estimated change and the estimates from Russell et al. (2007) [1] and AHRQ (2013) on the excess hospital costs of LBW babies, we calculate cost saving that arises from reduced prenatal smoking in response of GWLs. The reductions in the number of low birth weight babies attributed to GWL translate into substantial hospital cost savings. We estimate that the U.S. cost savings lie between 60.1 million dollars and 100.2 million dollars annually. These annual savings generate a present discounted value of savings over a 30 year horizon (0.03 discount rate) of 1.2 billion to 2.0 billion dollars.

## 2. Methods

### 2.1. Data

We extracted data from the 2013 Natality File that contains micro-level data on all births registered in the 50 United States, the District of Columbia, and New York City in calendar year 2013. The National Center for Health Statistics (NCHS) in conjunction with the Centers for Disease Control and Prevention (CDC) collects this data from individual records processed by each registration area, through the Vital Statistics Cooperative Program. Births are limited to births occurring within the United States. Births occurring to United States citizens outside of the United States are not included in the file. In total, the data set contains detailed information on 3,940,764 births occurring in the United States in 2013.

### 2.2. Dependent Variables

We created three dichotomous dependent variables representing different low birth weight categories. The first dependent variable, low birth weight, takes on a value of one if the baby was born with a weight of at least 1500 g but less than 2500 g, zero if the baby was born with a weight greater than or equal to 2500 g, and missing if the baby was born with a weight of less than 1500 g. The second dependent variable, very low birth weight, takes on a value of one if the baby was born with a weight of at least 1000 g but less than 1500 g, zero if the baby was born with a weight greater than or equal to 1500 g, and missing if the baby was born with a weight of less than 1000 g. Finally, the third dependent variable, extremely low birth weight, takes on a value of one if the baby was born with a weight of less than 1000 g, and zero otherwise.

### 2.3. Independent Variables

We created a rich set of independent variables to control for the determinants of low birth weight. Of particular importance to this research is whether or not the mother smoked during pregnancy. We created a dichotomous indicator equal to one if the mother smoked during pregnancy, and equal to zero otherwise.

We also created variables to control for characteristics of the mother including the mother’s age at pregnancy, the mother’s height and weight before pregnancy, and the number of pounds gained by the mother during pregnancy. We created a dichotomous indicator equal to one if the mother was married at the time of the birth and zero otherwise. In addition, we created mutually exclusive but all inclusive race/ethnicity indicators for the mother (Non-Hispanic Black, non-Hispanic American Indian or Alaskan Native, non-Hispanic Asian or Pacific Islander, Hispanic, and Non-Hispanic White—omitted as the reference category). Moreover, we created mutually exclusive but all inclusive indicators of the mother’s highest level of education (high school graduate, some college attendance, college graduate, graduate degree, and mother has less than a high school degree—omitted as the reference category).

We created variables to control for characteristics of the delivery and the baby. We created a dichotomous indicator equal to one if the baby was born in a hospital, and equal to zero otherwise. We created a dichotomous indicator equal to one if the baby was born via a cesarean delivery, and equal to zero otherwise. We created a dichotomous indicator for the gender of the baby equal to one if the baby was male, and equal to zero otherwise (i.e., female). We created a dichotomous indicator equal to one if the baby was born as part of a multiple birth, and equal to zero otherwise (i.e., singleton delivery). Finally, we created a continuous variable to reflect the birth order of the baby.

We also created mutually exclusive but all inclusive indicators for the source of payment for the delivery (Medicaid, out of pocket, other pay, and private insurance—omitted as the reference category). In addition, we created a dichotomous indicator equal to one if the mother receiving Women, Infants, and Children (WIC) benefits, and equal to zero otherwise. Finally, we created dichotomous birth month indicators to control for seasonality of birth.

## 3. Logit Results

We used logistic regressions to estimate the determinants of low birth weight babies. Table 1 contains the average marginal effects from three models. The average marginal effects for categorical variables are measured as discrete changes as the variables change from zero to one. The average marginal effects for continuous variables are measured as instantaneous rates of change (i.e., partial derivatives). Model 1 contains the estimates for the low birth weight regressions whereas Models 2 and 3 contain the estimates from the very low birth weight and extremely low birth weight equations, respectively.

Holding all other determinants of birth weight constant, we find cigarette use during pregnancy to have a positive and significant effect on all three low birth weight outcomes. The marginal effects found in Table 1 imply that the predicted probability of a baby being born with low birth weight, very low birth weight, and extremely low birth weight is 0.0430, 0.0032, and 0.0021 greater for a mother who smokes during pregnancy than for a mother who does not smoke during pregnancy, respectively.

With respect to the other results, the mother’s age is found to have a positive and significant effect on all three measure of low birth weight. Mothers who are Black are significantly more likely than whites to have all three measures of low birth weight babies. Mothers who are American Indian or Alaskan Native are significantly less likely to have a low birth weight baby than are Whites. Mothers who are Asian or Pacific Islanders are less likely to have an extremely low birth weight baby, but, more likely to have a low birth weight baby than are Whites. Hispanic mothers are less likely to have a very low birth weight and low birth weight baby than Whites. Mothers who are married are significantly less likely to have all three measures of low birth weight babies. Mothers who are high school graduates or have attended some college are more likely to have an extremely low birth weight baby, but, less likely to have a low birth weight baby than mothers who did not complete high school. Mothers who completed college or have a graduate degree are significantly less likely to have all three measures of low birth weight babies than mothers who did not complete high school. Birth order, pounds gained during pregnancy, higher pre-pregnancy weight, participation in WIC, and having a male baby are found to have a significant negative effect on all three measure of birth weight. Mother’s height is found to be negatively related to the probability of having a low birth weight baby, but positively related to having a very low birth weight or extremely low birth weight baby. Having a cesarean delivery or being part of a multiple birth increases the likelihood of all three low birth weight measures. The use of Medicaid to pay for the delivery increases the likelihood of all three low birth weight measures compared to insurance paying. Whereas, paying out of pocket increases extremely low birth weight babies, but, decreases low birth weight babies relative to paying with insurance. Using any other form of payment (other than Medicaid, out of pocket, and insurance) increases the likelihood of all three measures of low birth weight relative to insurance paying for the delivery. Finally, mothers who deliver in hospitals are more likely to have a very low birth weight or low birth weight baby than mothers who deliver elsewhere.

## 4. Predicted Effects of GWLs on Costs of Low Birth Weight Babies

In Table 2 we predict the effects of GWLs on hospital costs from low birth weight births. In our modeling, we express the extra hospital costs due to prenatal smoking as follows:

EHC = {N × pn + S × ps} × ehc
(1)

where: EHC = total extra hospital costs due to low birth weight babies; N = number of women giving birth who did not smoke during pregnancy; pn = probability of having a low birth weight baby for a woman who did not smoke during pregnancy; S = number of women giving birth who smoked during pregnancy; ps = probability of having a low birth weight baby for a woman who smoked during pregnancy; ehc = extra hospital cost per birth of a low birth weight baby. 

The causal effect of GWLs on extra hospital costs due to low birth weight babies can be expressed as:

∆EHC = {∆N × pn + ∆S × ps} × ehc
(2)

where: ∆N = the increase in the number of births to non-smoking women due to GWLs; ∆S = the decrease in the number of births to women who smoked during pregnancy due to GWLs; ∆N = −∆S.

We can therefore rewrite the equation for the causal effect of GWLs on extra hospital costs due to low birth weight babies as:

∆EHC = {−∆S × pn + ∆S × ps} × ehc
(3)

which simplifying to:

∆EHC = {ps − pn} × ∆S × ehc
(4)

Equation (4) shows that the decline in extra hospital costs due to prenatal smoking is equal to the number of women who do not smoke during pregnancy as a result of GWLs, times the reduction in risk of having a low birth weight baby due to becoming a non-smoker, times the average extra cost per low birth weight baby relative to a normal weight baby. Estimates of ps − pn come from Table 1. ∆S is estimated by taking the estimated number of births to women who smoked from the 2013 Natality data (334,400) and multiplying this times the 12%–20% reduction in smoking due to GWLs found for Canada by Huang et al. (2015). Estimates of ehc are taken from Russell et al. (2007) for low birth weight and extremely low birth weight births, and the authors’ computations using 2013 data from AHRQ for very low birth weight births, where all numbers are re-expressed in 2015 dollars using the consumer price index for medical expenditures.

Our projections in Table 2 indicate that a 12% reduction in smoking among pregnant women would reduce the number of low birth weight, very low birth weight, and extremely low birth weight babies by approximately 1726, 120, and 84 respectively, yielding first year cost savings of $40.97, $10.19, and $8.97 million dollars, respectively. A 20% reduction in smoking among pregnant women would reduce the number of low birth weight, very low birth weight, and extremely low birth weight babies by approximately 2876, 201, and 140, respectively, yielding first year cost savings of $68.3, $16.98, and $14.95 million dollars, respectively.

## 5. Discussion

To summarize, we find that GWLs would reduce hospital costs of low birth-weight babies due to prenatal smoking by $60.1 to $100.2 million per year. As this would be a recurrent benefit, the total expected benefits of this averted smoking would be far larger. For example, if we assume that the number of averted low birth-weight births remains in our estimated range of 1700–2900 per year, and using the 3% discount rate that is expected to be used in federal benefit-cost analyses, our annual cost savings would amount to $1.2 billion to $2.0 billion over a 30-year time horizon.

The 2009 FSPTCA (Family Smoking Prevention and Tobacco Control Act) gave the U.S. FDA authority to regulate the tobacco industry. The FDA conducted an economic impact analysis to assess the costs and benefits associated with proposed regulations as part of the rulemaking process required by the 2009 Family Smoking Prevention and Tobacco Control Act. Part of the regulation mandates graphic warning labels (GWLs) covering the top 50% of the front and back of cigarette packages. In 2011, the FDA issued regulations which required tobacco companies to add GWLs to cigarette packs. Chaloupka et al. (2015) [3] reviewed the FDA’s approach, offered suggestions for an improved analysis, and indicated that FDA’s analysis substantially underestimated the net benefits of GWLs by overestimating the costs and underestimating the benefits associated with the GWLs. The current study helps round out the need for better estimates of population-level benefits of FDA regulations intended to reduce smoking. While its estimates specifically relate to projected effects of GWLs, they will also be valuable for benefit-cost analyses of other rules that could be expected to reduce prenatal smoking, especially because they are up-to-date and population-based as is required for estimates used in federal benefit cost analyses. There is reason to believe that our estimates do not capture all the potential benefits from GWLs generated smoking reductions of prenatal maternal smoking. First, we do not consider other short term cost savings associated with the adoption of GWLs. For example, there are short term costs related to lost productivity by the parents. Having a LBW infant often results in changes in maternal employment and family income. Many mothers of LBW infants either postponed returning to work, reduced the number of hours they worked, or left the labor market after the birth of preterm or LBW infants which resulted in decreased family income [9,10,11]. There are also long run costs of LBW babies which we do not consider. First, LBW babies have on average higher lifetime medical costs than normal weight babies. Second, there are nonmedical costs associated with LBW babies. These include special education programs, adverse labor market outcomes, and other costs for society [12,13,14,15]. All of this suggests that our benefit estimates of GWLs are lower than the true total benefits that arise from reductions in prenatal maternal smoking. 

This study has other limitations. This study assumes that the effect of GWLs in reducing maternal smoking during pregnancy is the same in magnitude as for the general population. This study uses estimates of the effects of GWLs on smoking prevalence among general population obtained from Huang et al. (2014) [7] to compute the cost savings of reduced LBW babies due to the reduction of maternal smoking during pregnancy in response to GWLs. Several studies have found that pregnant women respond more to tobacco control regulations, such as taxes and smoke-free air laws, than the general population [16,17]. GWLs are effective in reducing smoking, by strengthening intentions to quit [8], encouraging quit attempts [18,19,20,21], preventing relapse [22], and discouraging smoking initiation [19,20]. Therefore, GWLs could motivate pregnant women to quit [23]. Pregnancy provides a motivational trigger for women to consider potential harms of their smoking behaviors imposed on themselves and on their babies. GWLs could have a larger effect on reduced smoking for pregnant women than the general population. Therefore, by using estimates of GWL’s effect on smoking prevalence for general population to compute cost savings of GWLs for pregnant women, our estimates of cost savings for pregnant women appear to be conservative.

To the extent that pregnant women would respond more to graphic warning labels than the general population and no other short or longer term costs on LBW are included in our projections, our estimated cost savings of between $60.1 million to $100.2 million would be conservative estimates of the true effects of GWL on cost savings. Finally, our estimates of the effects of maternal smoking during pregnancy on the probability of having low birth weight babies are based on 2013 birth data. It is possible that the smoking behavior of pregnant women will change in the future which could affect future probabilities of having low birth weight babies and cost savings estimates.

## 6. Conclusions

The reductions in the number of LBW babies attributed to GWLs translate into substantial cost savings. We estimated the annual cost savings resulting from GWL lie between 60.1 million dollars and 100.2 million dollars which translates to a long term saving of between 1.2 and 2.0 billion dollars over a 30 horizon. Through decreased smoking by pregnant women, GWLs protect the health of newborns and lead to substantial cost savings for society.

## Figures and Tables

**Table 1:** Determinants of the probability of having a low, very low, or extremely low birth weight baby, expressed as marginal effects.

Independent Variables	Model 1	Model 2	Model 3
	Probability of Having a Baby with:		
	Low Birth Weight	Very Low Birth Weight	Extremely Low Birth Weight
Cigarette Use	0.043 (69.04)	0.003 (14.47)	0.0021 (10.71)
Mother’s Age	0.0005 (17.93)	0.00003 (2.88)	0.00006 (6.19)
Mother Black	0.035 (73.68)	0.006 (30.07)	0.007 (38.65)
Mother American Indian/Alaskan Native	−0.003 (−2.42)	0.0006 (1.11)	−0.0007 (−1.51)
Mother Asian/Pacific Islander	0.005 (7.97)	−0.00004 (−0.21)	−0.0004 (−2.13)
Mother Hispanic	−0.007 (−21.50)	−0.001 (−7.19)	0.00001 (0.10)
Mother Married	−0.01 (−34.34)	−0.002 (−17.65)	−0.002 (−20.44)
Mother High School Graduate	−0.001 (−3.18)	−0.00007 (−0.48)	0.0005 (3.82)
Mother Some College	−0.003 (−7.49)	−0.0002 (−1.01)	0.0004 (2.78)
Mother College Degree	−0.012 (−22.87)	−0.001 (−8.06)	−0.001 (−7.39)
Mother Graduate Degree	−0.012 (−20.24)	−0.002 (−8.43)	−0.002 (−8.75)
Birth Order of Baby	−0.002 (−24.55)	−0.0002 (−7.01)	−0.0005 (−13.39)
Pounds Gained During Pregnancy	−0.002 (−155.04)	−0.0004 (−81.51)	−0.0005 (−99.55)
Pre-Pregnancy Weight	−0.0004 (−108.05)	−0.00005 (−38.68)	−0.00005 (−36.69)
Women Infants and Children	−0.004 (−12.10)	−0.002 (−16.21)	−0.003 (−27.10)
Male	−0.012 (−44.49)	−0.0004 (−3.62)	−0.0003 (−3.49)
Mother’s Height	−0.002 (−34.49)	0.00006 (2.87)	0.00007 (3.73)
Cesarean Delivery	0.028 (87.40)	0.011 (81.08)	0.006 (54.45)
Part of Multiple Birth	0.491 (289.26)	0.102 (72.90)	0.102 (66.25)
Medicaid	0.006 (16.16)	0.0009 (6.08)	0.0006 (4.26)
Out of Pocket	−0.001 (−1.98)	0.000005 (0.02)	0.0008 (2.87)
Other Method of Payment	0.003 (5.02)	0.0009 (3.38)	0.0007 (2.91)
In Hospital	0.027 (23.93)	0.001 (2.06)	−0.0003 (−0.55)
February	−0.0008 (−1.22)	−0.0004 (−1.53)	−0.0003 (−1.46)
March	−0.001 (−2.11)	−0.0002 (−0.81)	0.0004 (1.52)
April	−0.0003 (−0.49)	0.0002 (0.61)	0.0005 (2.28)
May	−0.001 (−2.06)	0.0001 (0.42)	0.0006 (2.46)
June	0.00007 (0.11)	0.0003 (1.37)	0.0003 (1.23)
July	−0.0008 (−1.33)	−0.0002 (−1.13)	−0.0001 (−0.68)
August	−0.002 (−3.07)	−0.0005 (−2.22)	−0.0004 (−2.05)
September	−0.002 (−3.36)	−0.0001 (−0.63)	−0.0002 (−1.05)
October	−0.0005 (−0.80)	−0.000006 (−0.02)	−0.0004 (−1.69)
November	0.0005 (0.75)	0.0002 (0.70)	−0.0003 (−1.22)
December	−0.00007 (−0.01)	−0.0001 (−0.51)	−0.0004 (−1.89)

Note. Z-statistics are in parentheses. The critical values for the z-statistics are 2.58 (2.33), 1.96 (1.64), and 1.64 (1.28) at the 1%, 5%, and 10% significance levels, respectively, based on a 2-tailed (1-tailed) test.

**Table 2:** Projected decline in hospital costs from low birth weight births due to implementing GWLs in the U.S.

Prenatal Smoking		Estimated Decline in Prenatal Smoking Due to GWLs		Derivation
		12%	20%	
1	Estimated reduction in number of women who smoke while pregnant due to GWLs	40,128	66,880	Baseline of 334,400 births to women who smoke from 2013 Natality data, times 12% or 20%
	**Low Birth Weight**			
2	Decline in probability of LBW births due to deterred smoking	0.043	0.043	Table 1
3	Decline in number of LBW births	1726	2876	Line 1 times line 2
4	Average cost per LBW birth	$23,746	$23,746	See discussion in text
5	Reduction in hospital costs from LBW births due to reduced prenatal smoking	$40,973,818	$68,289,697	Line 3 times line 4
	**Very Low Birth Weight**			
6	Decline in probability of VLBW births due to deterred smoking	0.003	0.003	Table 1
7	Decline in number of VLBW births	120.38	200.64	Line 1 times line 6
8	Average cost per VLBW birth	$84,627	$84,627	See discussion in text
9	Reduction in hospital costs from VLBW births due to reduced prenatal smoking	$10,187,737	$16,979,651	Line 7 times line 8
	**Extremely Low Birth Weight**			
10	Decline in probability of ELBW births due to deterred smoking	0.0021	0.0021	Table 1
11	Decline in number of ELBW births	84.27	140.45	Line 1 times line 10
12	Average cost per ELBW birth	$106,448	$106,448	See discussion in text
13	Reduction in hospital costs from ELBW births due to reduced prenatal smoking	$8,970,245	$14,950,409	Line 11 times line 12

GWLs: Graphic Warning Labels; LBW: Low Birth Weight; VLBW: Very Low Birth Weight.
